# Temozolomide Resistance in Glioblastoma Cell Lines: Implication of MGMT, MMR, P-Glycoprotein and CD133 Expression

**DOI:** 10.1371/journal.pone.0140131

**Published:** 2015-10-08

**Authors:** Gloria Perazzoli, Jose Prados, Raul Ortiz, Octavio Caba, Laura Cabeza, Maria Berdasco, Beatriz Gónzalez, Consolación Melguizo

**Affiliations:** 1 Institute of Biopathology and Regenerative Medicine (IBIMER), Granada, Spain; 2 Biosanitary Institute of Granada (ibs.GRANADA), SAS-Universidad de Granada, Granada, Spain; 3 Department of Health Science, University of Jaén, Jaén, Spain; 4 Cancer Epigenetics and Biology Program, Bellvitge Biomedical Research Institute, L'Hospitalet de Llobregat, Barcelona, Spain; 5 Service of Medical Oncology, San Cecilio Hospital, Granada, Spain; University Hospital of Navarra, SPAIN

## Abstract

**Background:**

The use of temozolomide (TMZ) has improved the prognosis for glioblastoma multiforme patients. However, TMZ resistance may be one of the main reasons why treatment fails. Although this resistance has frequently been linked to the expression of O6-methylguanine-DNA methyltransferase (MGMT) it seems that this enzyme is not the only molecular mechanism that may account for the appearance of drug resistance in glioblastoma multiforme patients as the mismatch repair (MMR) complex, P-glycoprotein, and/or the presence of cancer stem cells may also be implicated.

**Methods:**

Four nervous system tumor cell lines were used to analyze the modulation of MGMT expression and MGMT promoter methylation by TMZ treatment. Furthermore, 5-aza-2’-deoxycytidine was used to demethylate the MGMT promoter and O(6)-benzylguanine to block GMT activity. In addition, MMR complex and P-glycoprotein expression were studied before and after TMZ exposure and correlated with MGMT expression. Finally, the effect of TMZ exposure on CD133 expression was analyzed.

**Results:**

Our results showed two clearly differentiated groups of tumor cells characterized by low (A172 and LN229) and high (SF268 and SK-N-SH) basal MGMT expression. Interestingly, cell lines with no MGMT expression and low TMZ IC_50_ showed a high MMR complex expression, whereas cell lines with high MGMT expression and high TMZ IC_50_ did not express the MMR complex. In addition, modulation of MGMT expression in A172 and LN229 cell lines was accompanied by a significant increase in the TMZ IC_50_, whereas no differences were observed in SF268 and SK-N-SH cell lines. In contrast, P-glycoprotein and CD133 was found to be unrelated to TMZ resistance in these cell lines.

**Conclusions:**

These results may be relevant in understanding the phenomenon of TMZ resistance, especially in glioblastoma multiforme patients laking MGMT expression, and may also aid in the design of new therapeutic strategies to improve the efficacy of TMZ in glioblastoma multiforme patients.

## Introduction

Glioblastoma multiforme (GBM), the most common astrocytic tumor, representing about 65% of all adult nervous system tumors, is characterized by a high aggressiveness, with an average survival period of less than 15 months [1–4]. Current treatment options, including surgery, radiation therapy, and chemotherapy [2], shows a limited response due to blood-brain barrier (BBB) protection, the absence of a lymphatic drainage system, and development of drug resistance [5]. In this context, a better understanding of GBM resistance mechanisms may lead to the development of new therapeutic strategies.

Temozolomide (TMZ), a second-generation imidazotetrazine lipophilic prodrug, has improved the prognosis for GBM patients because it can cross the BBB and induce glioblastoma cell death by introducing alkyl groups into DNA [6]. Temozolomide is highly stable at stomach acid pH but spontaneously undergoes hydrolysis to the active metabolite MTIC [5-(3-dimethyl-1-triazenyl)imidazole-4-carboxamide] at physiological pH, thus releasing the drug's activity in the tumor tissue [7]. The drug forms O6-methylguanine adducts that introduce mispairs with thymine, which cannot be repaired thereby inducing the formation of single- and double-strand DNA breaks and triggering apoptosis and senescence mechanisms in glial cells [8,9]. However, the presence of some drug-resistance mechanisms appears to be responsible for the therapeutic failure of TMZ in GBM patients.

Two candidates, namely O6-methlyguanine-DNA-methyltransferase (MGMT) and the mismatch repair (MMR) system, have been associated with ineffective GBM therapy, although their relationship is not yet clear. The MGMT repair protein protects the cellular genome from the mutagenic effects of alkylating agents such as TMZ by removing the O6-alkylguanine DNA adduct. This adduct is transferred from the alkyl group to one of its own cysteine residues and normal guanine is restored [10], thereby reducing the effect of TMZ. MGMT promoter methylation status is responsible for regulating MGMT expression and has been correlated with increased GBM patient survival [11] although subsequent studies suggested that this association is inconclusive [12]. However, MMR is critical for the maintenance of replication fidelity and for inducing appropriate cellular responses to DNA damage [13]. The functions of this protein complex, which includes the proteins codified by the genes MLH1, MSH2, MLH3, MLH6 and PMS2 [14], are not fully known. Moreover, an MMR deficiency has been correlated with genetic instability in colorectal cancer [9,14]. In GBM, TMZ treatment induces DNA lesions such as O6-MeG which cannot be repaired by MGMT, with the MMR system causing double-strand DNA breaks and apoptosis [15]. As such, the MMR complex must work properly in order for TMZ to carry out its cytotoxic function. Indeed, Goellner et al. [16] showed a relationship between TMZ resistance and MMR failure in GBM patients. In addition, some authors have attempted to correlate TMZ resistance in GBM patients to the presence of P-glycoprotein (P-gp) acts as an efflux pump that expels the drug from the cell, thus reducing its effectiveness, in the membrane of tumor cells [10,17]. This phenomenon, known as multidrug resistance phenotype (MDR), results in the survival of tumor cells despite drug treatment and the subsequent failure of chemotherapy against several types of tumor [18]. Finally, Cancer Stem Cells (CSC) have also been related to tumor resistance to chemotherapy and radiotherapy. CD133 expression may be used to detect and evaluate the population of CSCs inside certain tumors, including GBM [19]. However, although the presence of high amounts of CSCs in tumors appears to be associated with a worse prognosis and a reduced response to treatment, the exact correlation is still unclear [20].

The aim of this study was to analyze the relevance of MGMT, the MMR system and P-gp in the development of resistance against TMZ in tumor cell lines A172, LN229, SF268 and SK-N-SH. Interestingly, we have demonstrated a significant correlation between MGMT and MMR complex expression such that cell lines with no MGMT expression and a low TMZ IC_50_ presented high MMR complex levels. In contrast, cell lines with a high MGMT expression and a high IC_50_ against TMZ did not express the MMR complex. In addition, neither P-gp nor CD133 expression appeared to play a relevant role in the TMZ resistance phenomenon of these cell lines. Thus, the status of the MMR complex could be related to MGMT activity in GBM patients as both are related to TMZ resistance. This connection could have clinical importance in terms of explaining TMZ resistance in GBM patients and, therefore, the differences in their responses to treatment.

## Methods

### Cell lines

A172, LN229 (from American Type Culture Collection: CRL-1620™ and CRL-2611™, respectively) and SF268 (from Scientific Instrument Center, Granada University) human glioblastoma cell lines and the SK-N-SH human neuroblastoma cell line (from American Type Culture Collection: HTB-11™) were grown in Dulbecco’s Modified Eagle’s Medium (Sigma, St. Louis, MO, USA), supplemented with 10% fetal bovine serum (FBS) and 1% antibiotics (penicillin and streptomycin). Cells were maintained in monolayer culture at 37°C under a humidified atmosphere containing 5% CO_2_.

### Drugs and reagents

Temozolomide (TMZ), 5-aza-2’-deoxycytidine (5Aza) and O(6)-benzylguanine (O6-BG) were purchased from Sigma-Aldrich. Aliquots of TMZ dissolved in DMSO (20 mg/ml) and prepared in serum-free culture medium (10 mg/ml) were protected from light and stored at –20°C. O6-BG stocks were dissolved in methanol (10 mg/ml) and stored at room temperature.

### *In vitro* drug treatments

Temozolomide treatment of all tumor cell lines comprised a double cycle (3 days of drug exposure followed by 3 days without drug) using the previously determined IC_50_ dose. Cell lines exposed to the first and second TMZ cycle (named -1C and -2C, respectively) were subsequently subjected to further studies at the IC_50_ for TMZ. 5-Aza was used in de-methylation studies at a concentration of a 30 μM for A172 and LN229 and 10 μM for SF268 and SK-N-SH. In addition, SF268 and SK-N-SH cell lines were exposed to 30 μM O6-BG prior to TMZ treatment.

### Cytotoxicity assays

Cell lines exposed to TMZ (with or without 5-Aza or O6-BG pre-treatment) were grown in 24-well plates (Sigma) under standard culture conditions for 6 days. Cytotoxicity was determined using the sulphorhodamine-B (SRB) method. Briefly, the cells were fixed with 10% trichloroacetic acid for 20 min at 4°C then washed three times with water. After 24 hours, cells were stained for 30 min at room temperature with 0.4% SRB dissolved in 1% acetic acid and then washed three times with 1% acetic acid. The plates were air-dried and the dye solubilized with 300 ml/well of 10 mM Tris base (pH 10.5) for 10 min on a shaker. The optical density of each well was measured spectrophotometrically using a Titertek multiscan colorimeter (Flow, Irvine, California) at 492 nm.

### Methylation-specific PCR analysis

DNA was extracted from culture cells using the *QIAamp DNA Mini Kit* (EpiTect Bisulfite kit, Qiagen, Maryland, USA) in accordance with the manufacturer's standard recommendations. Thus, 2 μg of DNA from each cell line was denatured, modified, and purified using the EpiTect Bisulfite kit (Qiagen, Maryland, USA). The MGMT promoter CpG islands methylation status of different cell lines was based on chemical modification of unmethylated cytosine with bisulfite to uracil. Methylation-specific PCRs (MSP) were performed using specific primers for either methylated or unmethylated DNA in the MGMT promoter. Primer sequences for MGMT were 5'-TTTGTGTTTTGATGTTTGTAGGTTTTTGT-3' (forward primer) and 5'-AACTCCACACTCTTCCAAAAACAAAACA-3' (reverse primer) for the unmethylated (UM) reaction and 5'-TTTCGACGTTCTAGGTTTTCGC-3' (forward primer) and 5'-GCACTCTTCCGAAAACGAAACG-3' (reverse primer) for the methylated (M) reaction. Agarose electrophoresis visualization by ethidium bromide and UV illumination was performed after PCR.

### High-resolution MGMT methylation analysis

The high-resolution MGMT methylation analysis of bisulfite samples was performed using high-sensitive SYBR® Green (KapaBiosystems, Boston, USA) at the Center for Genomics and Oncological Research (GENYO, Granada). The reaction was conducted using an Eco Real-Time PCR System (Illumina, CA, USA) and data were analyzed using the Eco Real-Time PCR System v4.0 software. Methylated EpiTect Control DNA, methylated and unmethylated EpiTect Control DNA, (Qiagen, Madrid, Spain) were used for the methylation curve, with methylated-unmethylated ratios of 0, 0.25, 0.5, 0.75, and 1. All samples and the methylation curve were analyzed using a pair of primers for the specific region.

### mRNA expression analysis

Total RNA was extracted using an RNA purification system (RNeasy, Qiagen). Reverse transcription-PCR was performed with 1.5 μg of isolated total RNA and synthesized to cDNA in a 20 μl reaction system using reverse transcriptase (Promega) with oligo-dT primers according to the manufacturer’s instructions. cDNA was used to determine MGMT expression before and after 5Aza treatment with the following primers: Fw 5'- TCACGGCCAGTCCTCCGGAG -3' and Rw 5'- GTTCCCCGTGCCGGCTCTTC -3'. PCR was performed under classical conditions with a melting temperature of 58°C. Amplified products were separated by electrophoresis on a 3% agarose gel and visualized under UV illumination. In addition, cDNA was amplified by real-time PCR in 96-well plates using an Applied Biosystem 7500 system (Applied Biosystems, Life Technologies). The total reaction volume of 20 μl contained 20 ng cDNA, 1xTAqMan Universal PCR Mastermix (Applied Biosystems, Life Technologies), and 1x TaqMan Gene Expression assay (Applied Biosystems, Life Technologies). The PCR was run at 50°C for 2 min, 95°C for 10 min, and 40 cycles of 95°C for 15 s and 60°C for 60 s. Each sample was analyzed using the TaqMan Gene Expression Assays *MGMT* (Hs01037698_m1 Applied Biosystems, Life Technologies), *ABCB1* (Hs00184500_m1 Applied Biosystems, Life Technologies) as well as an endogenous control GADPH (Applied Biosystems, Life Technologies). For MMR genes (Table 1), real-time PCR was carried out as described previously using Sybergreen reagent according to the manufacturer's instructions (Takara, Clontech Laboratories, Inc. USA). Ct-values for all samples were determined automatically with the default setting using StepOne Software V2.0 (Applied Biosystems, Life Technologies). Gene expression levels were calculated using the ΔCt method: ΔCt = mean value Ct (mRNA reference) − mean value Ct (mRNA of interest). Normalised ΔCT (delta cycle threshold) values were obtained by subtracting the Ct for GADPH from that for the gene of interest. The relative mRNA expression of the gene of interest corresponded to the value 2^ΔCt^.

**Table 1:** Primer sequences used to analyze *MMR*.

Primer name	Sequence
MLH1-F	TTC GTG GCA GGG GTT ATT CG
MLH1-R	GCC TCC CTC TTT AAC AAT CAC TT
MSH2-F	GCT GGA AAT AAG GCA TCC AAG G
MSH2-R	CAC CAA TGG AAG CTG ACA TAT CA
MSH3-F	TGG AAA ATG ATG GGC CTG TTA AA
MSH3-R	AGA CAT TCC CAG ATC ACT TCC T
MSH6-F	AGC TTA AAG GAT CAC GCC ATC
MSH6-R	AAG CAC ACA ATA GGC TTT GCC
PMS2-F	GAA GGT TGG AAC TCG ACT GAT
PMS2-R	CGC ACA GGT AGT GTG GAA AA
GADPH-F	TGC ACC ACC AAC TGC TTA GC
GADPH-R	GGC ATG GAC TGT GGT CAT GAG

All primers are written in a 5' to 3' direction and grouped according to pairs. F, forward primer; R, reverse primer.

### Western blot analysis

Cells were washed twice with phosphate-buffered saline (PBS) and lysed with a lysis buffer (Trizma base 50 mM, sacarose 0.25 mM, EDTA 5 mM and triton X-100 0.5%, pH 7.4). Protein concentration was determined using Bradford Reagent (Bio-Rad) after sonication. Thus, 25 μg of protein was electrophoretically separated by 12% SDS-PAGE and transferred to nitrocellulose membranes. These membranes were blocked for 30 min at room temperature in 5% (w/v) milk powder in PBS containing 0.1% Tween 20, co-incubated overnight at 4°C with the primary antibodies (MGMT 1:200, β-actin 1:10000 dilution), washed three times with 0.1% Tween 20 in PBS, and incubated for 1 h with a horseradish peroxidase-conjugated (HRP) goat anti-mouse secondary antibody 1:2500 (Santa Cruz Biotechnology). Proteins were visualized using the ECL system (Amersham Biosciences, USA) in the LAS-4000 mini equipment. Further analysis, as well as image processing and quantification of the bands, was performed using the program ImageQuant Las-4000. MGMT expression was normalized relative to the β-actin level of the tumor.

### Flow cytometry analysis

The cell-cycle distribution was determined by flow cytometry. Thus cells were treated with TMZ (IC_50_ doses for 120 hour), harvested, and fixed in 70% (v/v) cold ethanol for 20 minutes. They were then pelleted, washed once with PBS and resuspended in propidium iodide (PI) solution (50 μg/mL PI, 0.5 mg/mL RNase staining buffer) for 30 min in the dark. Data were collected and analyzed using the Cellfit program with a FACScan flow cytometer (FACSCanto II Cytometer; BD Biosciences, San Jose, CA). In addition, CD133 marker for cancer stem cells were also studied by FACScan. Cells were removed from culture using a nonenzymatic cell dissociation solution (Sigma-Aldrich, Madrid, Spain) and washed with PBS. Approximately 2 × 10^5^ cells were incubated with primary CD133 antibody directly coupled to phycoerythrin (Miltenyi Biotec, Bergisch Gladbach, Germany) for 15 minutes in the dark at room temperature. Isotypic controls were used to establish the right gating.

### Statistical analysis

Statistical evaluations were carried out using SPSS statistical software, version 16.0 (SPSS Inc., Chicago, IL, USA). The level of statistical significance was set at p < 0.05 for all tests. Experimental data were expressed as mean ± standard deviation (SD) and the results compared using Student’s t-test.

## Results

### Temozolomide IC_50_ in tumor cell lines

**Fig 1:**
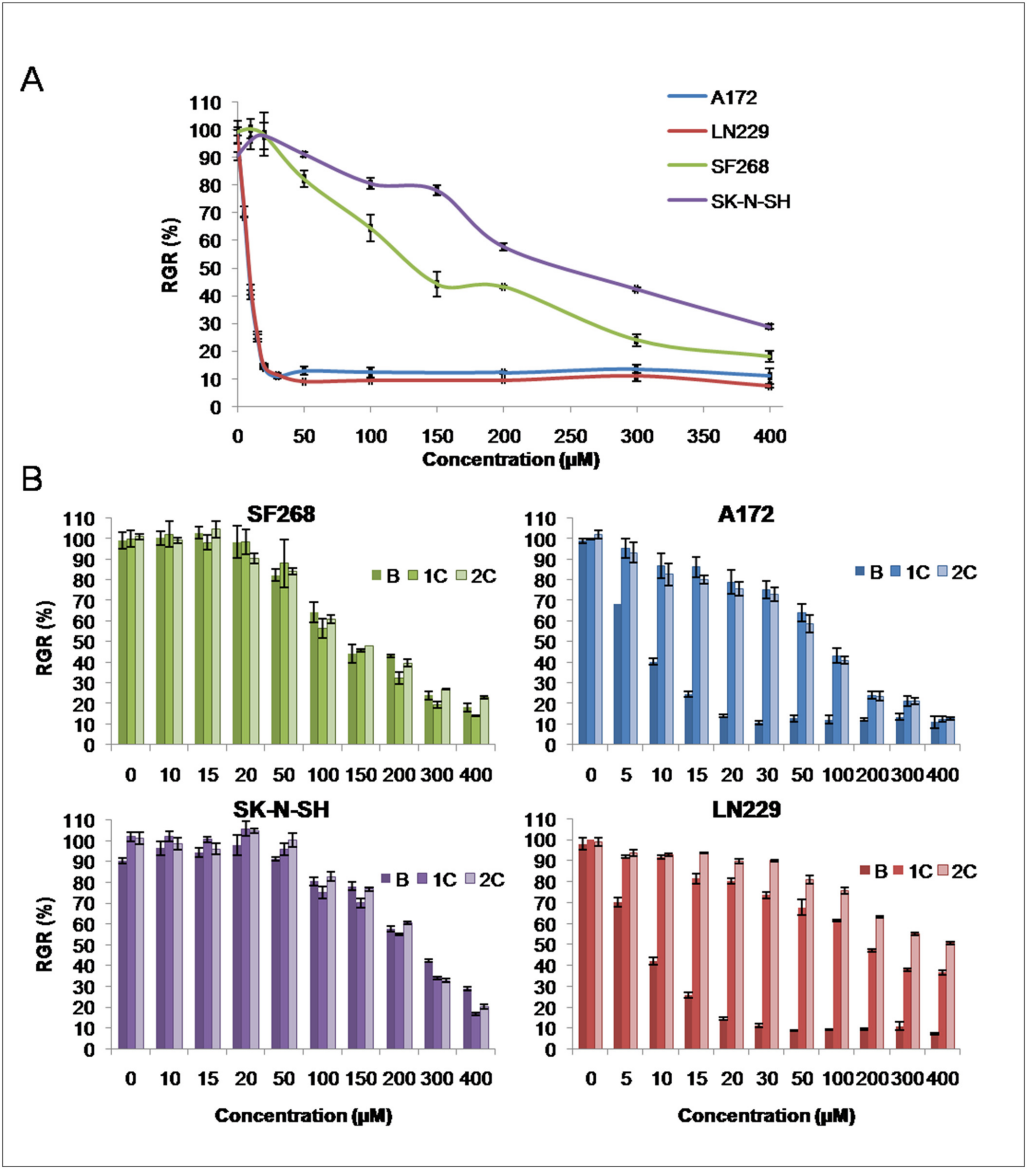
Temozolomide toxicity. A) Relative growth rates (RGR) for tumor cell lines treated with TMZ. B) Relative growth rates for tumor cell lines before and after TMZ cycling treatment (B: basal cells; 1C: first TMZ cycle; 2C: second TMZ cycle). SF268 and SK-N-SH lines did not reveal any IC_50_ variation, whereas A172 and LN229 showed a large increase in TMZ IC_50_. All data represent the mean value ± SD of triplicate cultures.

Determination of the IC_50_ for TMZ in different cell lines gave values ranging from 14.1 to 234.6 μM that fell into two clearly differentiated groups (Fig 1A): cell lines with low IC_50_ values (< 50 μM), which included A172 (14.1 ± 1.1 μM) and LN229 cells (14.5 ± 1.1 μM), and those with high IC_50_ values (> 100 μM), which included SF268 (147.2 ± 2.1 μM) and SK-N-SH cells (234.6 ± 2.3 μM). Analysis of the modulation of TMZ toxicity using two cycles of treatment demonstrated that the TMZ IC_50_ increased significantly in cell lines with a low basal IC_50_, whereas no modulation was observed in cell lines with a high basal TMZ IC_50_ (Fig 1B). In fact, LN229 and A172 cell lines reached a TMZ IC_50_ 35.3 (547.4 ± 2.6 μM; p = < 0.0001) and 5.5 times (77.5 ± 1.8 μM; p = 0.0028) higher, than their respective basal values (Fig 1B).

### Cell cycle and TMZ treatment

**Fig 2:**
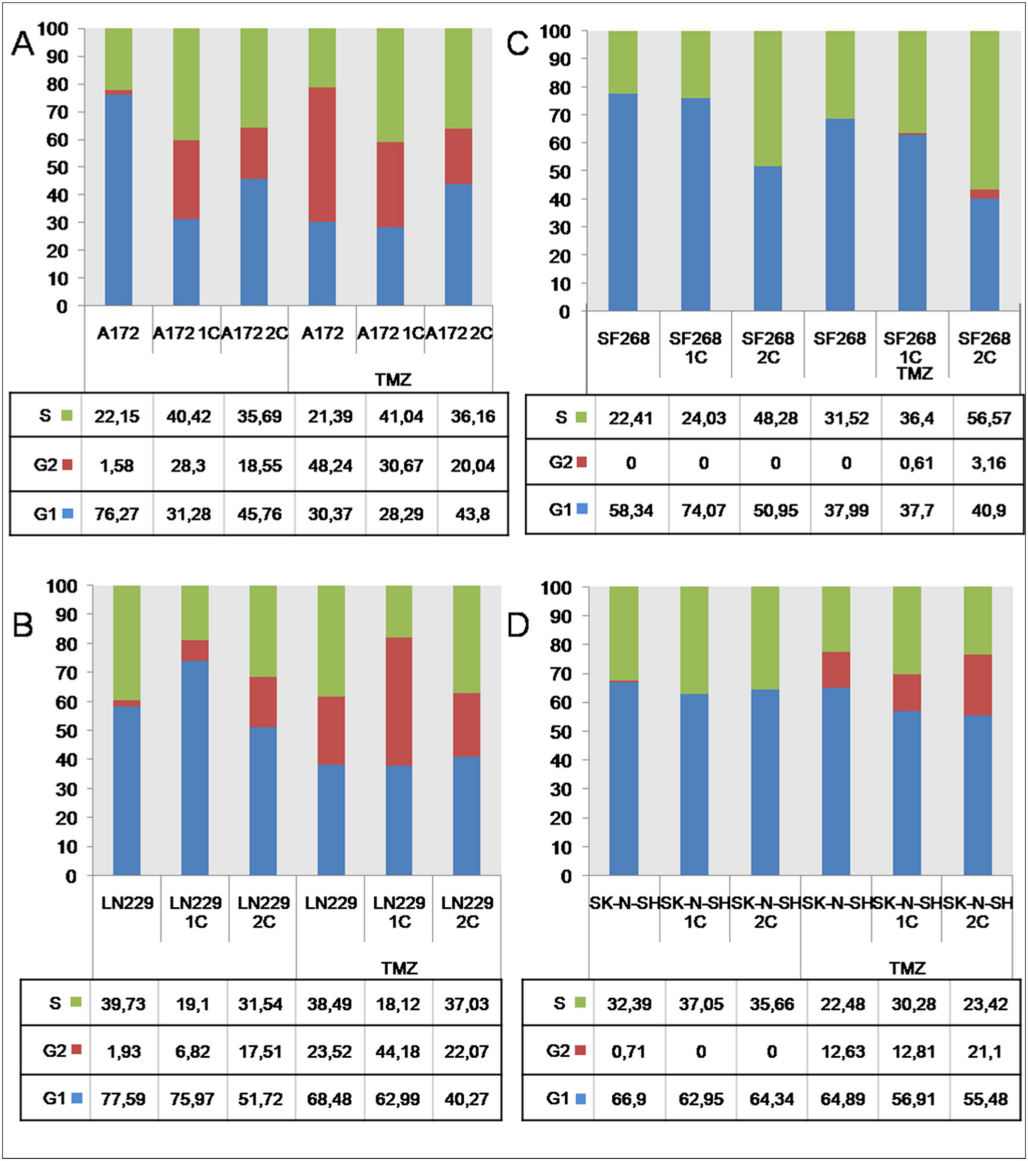
Cell cycle modulation induced by TMZ treatment. Modulation of the cell cycle (G1, S and G2) by TMZ treatment in A172 (A), LN229 (B), SF268 (C) and SK-N-SH (D) cell lines. The number of cells in the G1-, S or G2-phase is given as percentages of the total cell population.

The cell cycle of the tumor cell lines were studied before and after TMZ treatment using the previously determined values for TMZ IC_50_. As shown in Fig 2A, cell-cycle phases in the A172 cell line (76.27% in G1, 22.15% in S, and 1.58% in the G2/M phase) were modified by TMZ treatment which induced a significant decrease in the G1 phase (30.37%) and an increase in the G2/M and S phases (48.24% and 21.39%, respectively). Similar results were observed for the LN229 cell line (Fig 2B) with TMZ exposure inducing a reduction in the G1 phase (from 58.34% to 37.99%) and an increase in the G2/M phase (from 1.93% to 23.53%). No significant modification was observed in the S phase. In the SF268 cell line (Fig 2C), TMZ treatment induced a significant increase in the S phase (from 22.41% to 31.52%) at the expense of the G1 phase, whereas no modifications were observed in the G2/M phase. In the SK-N-SH cell line (Fig 2D), TMZ treatment caused cells to appear in the G2/M phase (21.1%), a decrease in the S phase from 32.39% to 22.48%, and a minor variation in the G1 phase (from 66.9% to 64.89%). These cells were also subjected to two TMZ cycles in order to observe modulation in the cell cycle distribution. SF268 cells (Fig 2C) showed an S phase increase (from 22.41% to 58.57%) and a G1 reduction (from 77.59% to 40.27%). There was almost no population in the G2/M phase during TMZ treatment for this cell line. In A172 cells (Fig 2D), TMZ treatment caused a decrease in the G2/M phase (20.4%) and increases in the S phase (from 21.39% to 36.16%) and the G1 phase (from 30.37% to 43.80%). For the rest of the cell lines, consecutive exposures to TMZ had a little effect on the behavior of the cell cycle.

### Modulation of MGMT promoter methylation by TMZ

**Fig 3:**
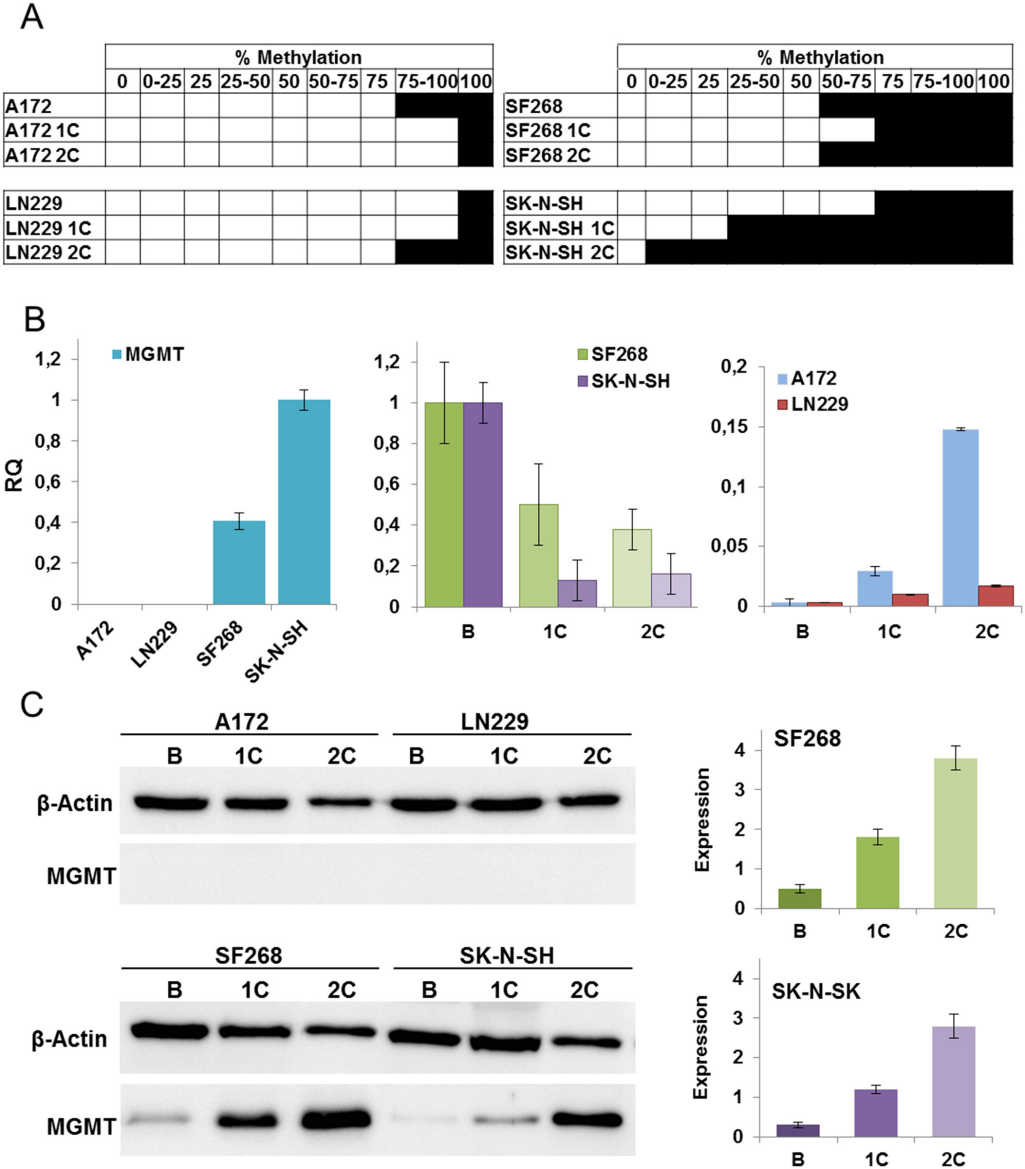
Analysis of MGMT promoter methylation and MGMT expression levels. A) Modulation of the MGMT promoter methylation percentage in tumor cell lines before and after TMZ treatment. B) Real-time PCR analysis of MGMT expression before and after TMZ treatment. Showing relative expression of MGMT compared with SK-N-H cell line. C) Western blot analysis of MGMT protein expression in tumor cell lines before and after TMZ treatment. Beta-actin expression was used as a control. The graphs on the right show the densitometry of the MGMT Western blot analysis in SF268 and SK-N-SH cell lines before and after TMZ treatment. B: basal cells; 1C: first TMZ cycle; 2C: second TMZ cycle. All data represent the mean value ± SD of triplicate cultures.

The MGMT promoter CpG island methylation percentage was determined before and after treatment of each cell line with TMZ (Fig 3A). The A172 and LN229 cell lines (with low TMZ IC_50_ values) showed a high percentage of promoter methylation, reaching 100% in the LN229 line and 75–100% in the A172 cell line. No significant changes were detected in either of these lines after TMZ treatment. In contrast, a lower percentage of promoter methylation was detected in untreated SF268 (50–75%) and SK-N-SH (75%) cell lines (with high TMZ IC_50_ values). After TMZ treatment, SK-N-SH showed a significant decrease to 0–25% after the second cycle of TMZ. However, no change in promoter methylation percentage was observed for the SF268 cell line.

### Modulation of MGMT expression after TMZ treatment

Real-time PCR analysis showed a significant level of MGMT expression in SF268 and SK-N-SH cell lines (with high TMZ IC_50_ values), whereas no MGMT expression was observed in the A172 and LN229 cell lines (with low TMZ IC_50_ values; Fig 3B). A significant decrease in MGMT expression after the second cycle of TMZ treatment was detected in SF268 (70%) and SK-N-SH cell lines (80%; Fig 3B). In contrast, the A172 and LN229 cell lines, which exhibit no basal MGMT expression, showed a slight increase in MGMT after TMZ administration but expressed at very low levels compared to both SF268 and SK-N-SH cell lines (Fig 3B).

The MGMT protein levels in cell lines before and after TMZ treatment were detected using the western blot technique. Our results demonstrated that MGMT expression in both SF268 and SK-N-SH cell lines (Fig 3C) increased approximately three-fold after TMZ treatment (Fig 3C). In contrast, MGMT protein expression in the A172 and LN229 cell lines was barely detectable despite TMZ treatment (data not shown; Fig 3C).

### Modulation of MGMT expression by 5Aza and O6BG treatment

**Fig 4:**
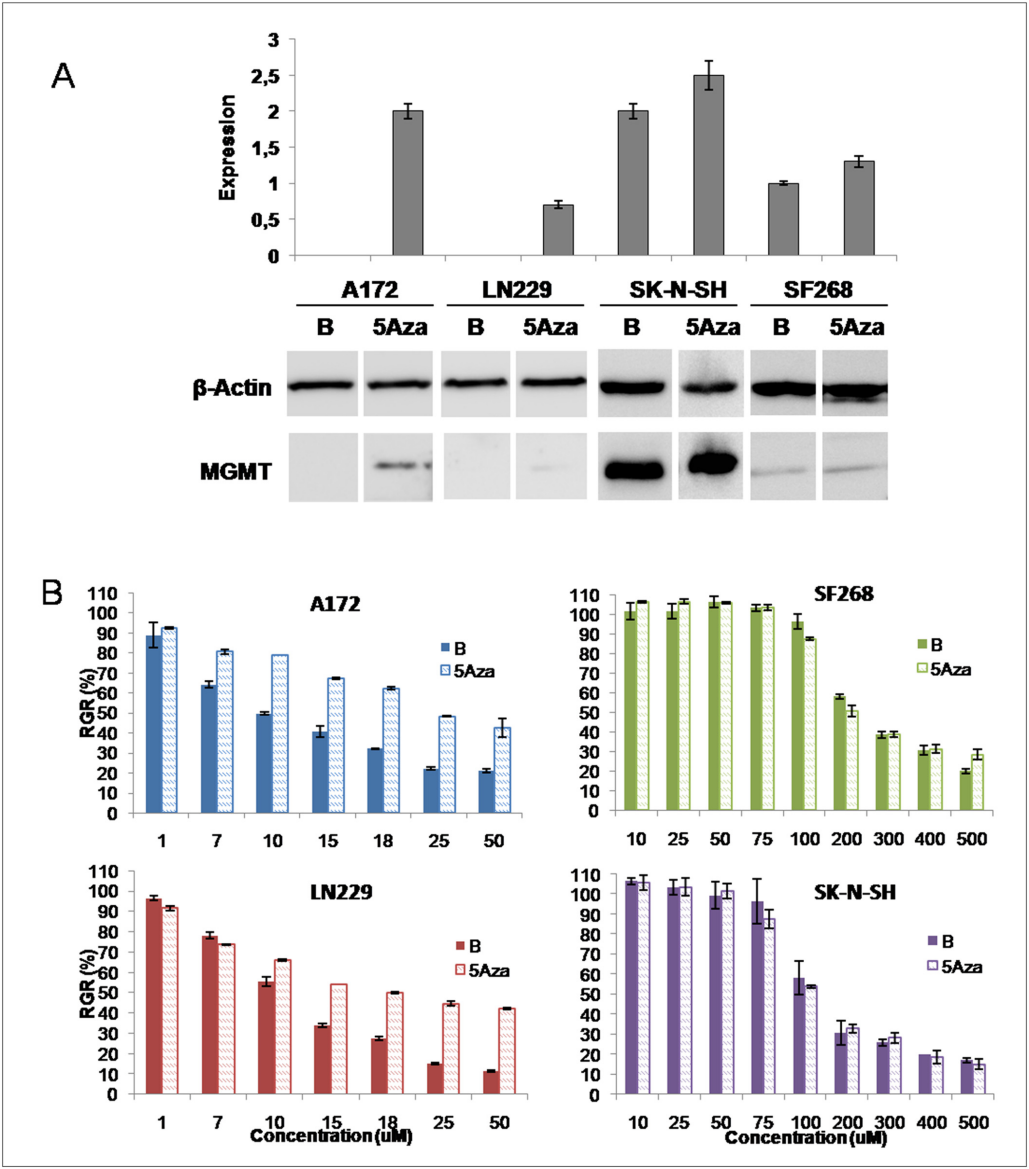
Modulation of MGMT expression by 5-Aza. A) Densitometric analysis of the effect of 5-Aza for each cell line showing relative expression of MGMT compared with beta-actin under each experimental condition. Western blot analysis of MGMT expression after 5-Aza exposure in tumor cell lines. Beta-actin expression was used as a control. B) Relative growth rates of tumor cell lines before and after 5-Aza exposure. B: basal cells, 5Aza: cell line treated with 5-Aza. All data represent the mean value ± SD of triplicate cultures.

To determinate the relevance of MGMT in the modulation of TMZ toxicity, 5-Aza was used to induce MGMT expression and O6-BG to block its activity. As shown in Fig 4A, 5-Aza treatment induced an increase in MGMT protein expression in both SF268 and SK-N-SH cell lines, which was quantified by densitometry (25% and 50%, respectively) whereas only, a slight modulation of MGMT expression was observed in A172 and LN229 cell lines (Fig 4A). In addition, cell lines exposed to 5-Aza were assayed to determine the variation in the TMZ IC_50_. As shown in Fig 4B, A172 and LN229 cell lines showed a significant increase in the TMZ IC_50_ (A172 increased from 14.1 ± 1.1 μM to 66.84 ± 1.6 μM; LN229 increased from 14.5 ± 1.1 μM to 66.93 ± 1.6 μM; p = < 0.0001 and p = < 0.0001, respectively) whereas, no significant variation in TMZ IC_50_ was observed for SF268 and SK-N-SH cell lines (142 ± 1.1 μM and 238 ± 1.1 μM, respectively; p = 0.3875; Fig 4B).

**Fig 5:**
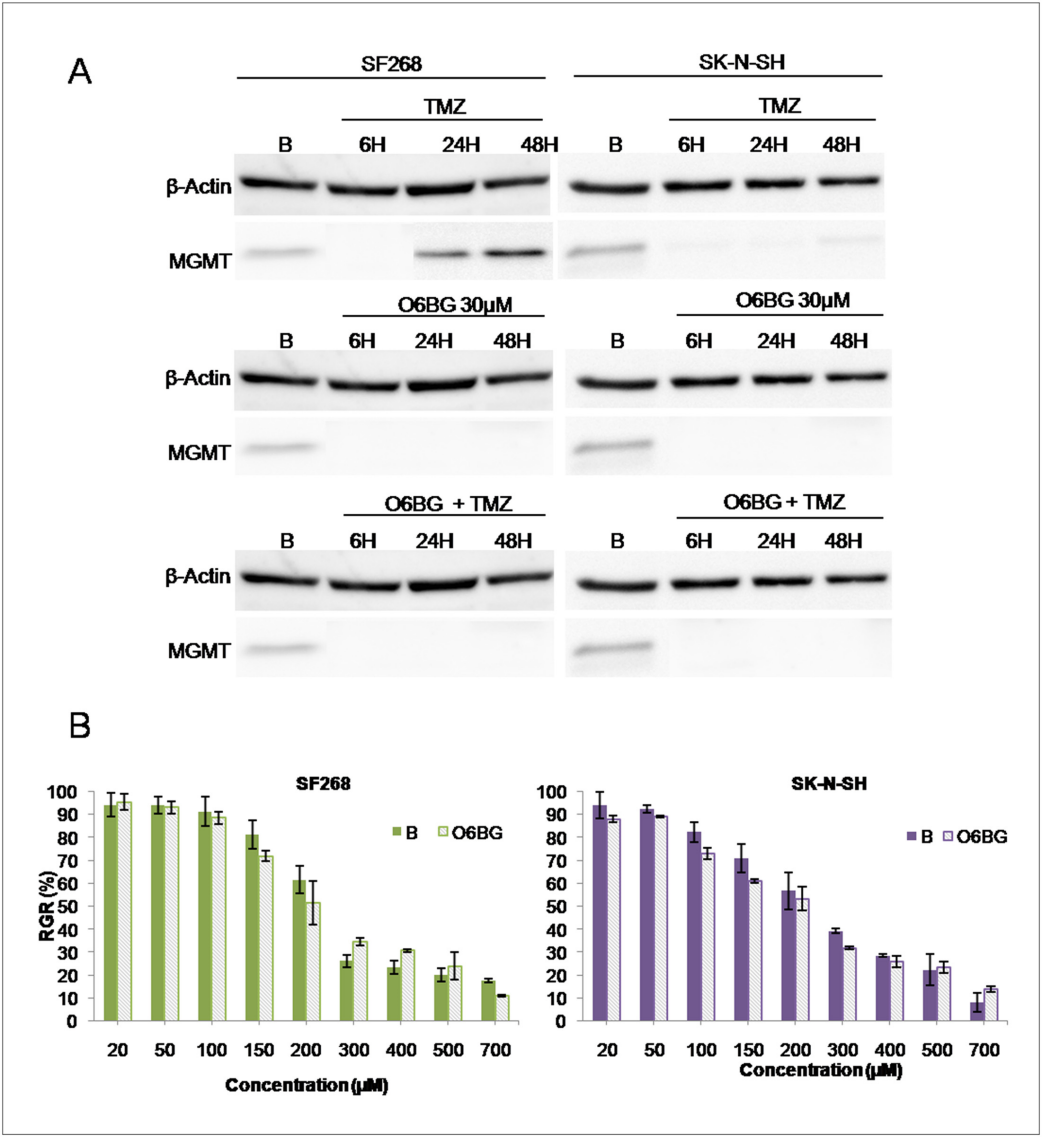
Modulation of MGMT expression by O6-BG in SF268 and SK-N-SH tumor cell lines. A) Western blot analysis of the MGMT expression after treatment with TMZ, O6-BG and both TMZ + O6-BG. B) Relative growth rates of SF268 and SK-N-SH cell lines with and without O6-BG treatment after TMZ treatment. B: basal cells, O6BG: cell line treated with O6-BG. All data represent the mean value ± SD of triplicate cultures.

O6-BG was used to silence MGMT only in those cells which expressed MGMT (SF268 and SK-N-SH). As shown in Fig 5A, O6-BG treatment completely inhibited MGTM expression in both SF268 and SK-N-SH cell lines even after exposure to TMZ treatment. Furthermore, cell lines treated with O6-BG were tested using cytotoxicity assays to determine any variation in the TMZ IC_50_. Interestingly, no significant changes in the TMZ IC_50_ value were detected compared to that for untreated cells (p = 0.098; Fig 5B).

### Modulation of MMR expression by TMZ treatment

**Fig 6:**
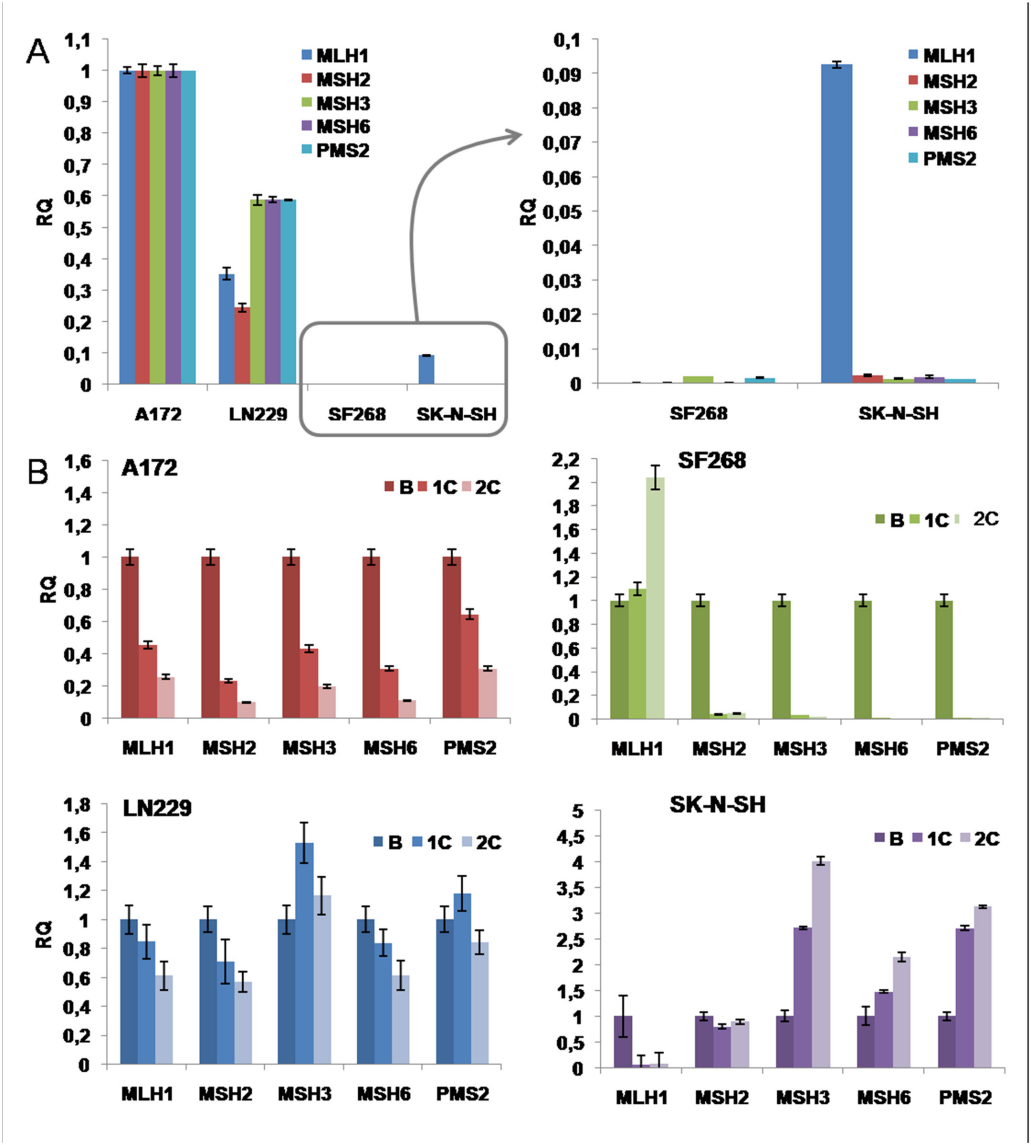
Modulation of MMR expression by TMZ treatment. A) Real-time PCR analysis of MMR gene expression levels (MLH1, MSH2, MSH3, MSH6 and PMS2) in tumor cell lines. B) Modulation of MMR gene expression levels after TMZ treatment in tumor cell lines. B: basal cells; 1C: first TMZ cycle; 2C: second TMZ cycle. All data represent the mean value ± SD of triplicate cultures.

Real-time PCR was used to determine the expression of five genes for the MMR system (MLH1, MSH2, MSH3, MSH6 and PMS2) in tumor cell lines. The highest level of MMR subunit expression was observed in A172 and LN229 cell lines, whereas such expression was practically undetectable in SF268 and SK-N-SH lines (Fig 6A). After TMZ treatment, the MSH2 and MSH6 subunits decreased in A172 (90%) and LN229 (40%) cell lines (Fig 6B) whereas, the SF268 cell line presented a decrease in the quantity of all MMR subunits (except MLH1; Fig 6B). For SK-N-SH, all MMR subunits increased in number (except MLH1, which was practically null; Fig 6B). Consequently, our results demonstrate that TMZ produced an overall decrease in most of the MMR complexes expressed, thus inducing a reduction in their effective functional capacity.

### Modulation of P-glycoprotein expression by TMZ treatment

**Fig 7:**
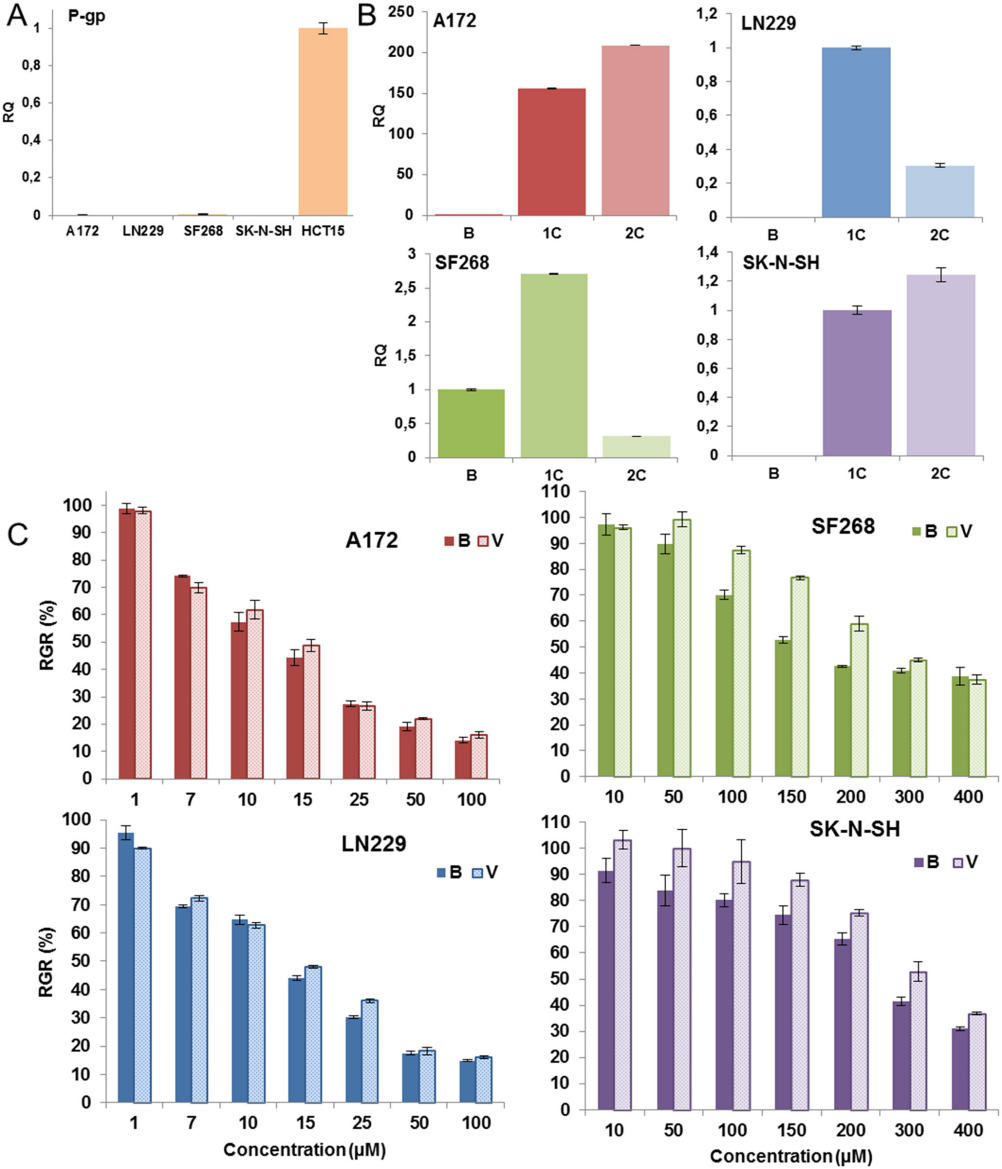
Modulation of P-gp expression by TMZ treatment. A) Real-time PCR analysis of P-gp expression levels in tumor cell lines. The TMZ-resistant HCT15 cell line was used as a control. B) Modulation of P-gp expression by TMZ treatment in tumor cell lines. C) Relative growth rates after exposure to TMZ in tumor cell lines before and after verapamil (V) treatment. B: basal cells; 1C: first TMZ cycle; 2C: second TMZ cycle. All data represent the mean value ± SD of triplicate cultures.

P-glycoprotein expression was determined by Western blot for all tumor cell lines. The resistant human colorectal cancer cell line HCT-15 which endogenously expresses of P-gp, was used as a control. As shown in Fig 7A, all tumor cell lines were negative for P-gp expression. TMZ treatment induced an increases in P-gp expression in all tumor cell lines, with only LN229 and SF268 cell lines showing a decrease after the second TMZ cycle (Fig 7B). To determine the relevance of P-gp expression as regards TMZ cytotoxicity, verapamil was used to block P-gp before determining the TMZ IC_50_. As shown in Fig 7B, cell lines with no MGMT expression (A172 and LN229) did not present any changes in TMZ IC_50_ after verapamil treatment (p = 0.3875), whereas cell lines with high levels of MGMT expression (SF268 and SK-N-SH) showed a significant increase in the value of TMZ IC_50_ (from 147.2 ± 2.1 μM to 276 ± 2.4 μM in SF268 and from 234.6 ± 2.3 μM to 318.2 ± 2.5 μM in SK-N-SH; p = 0.0379 and p = < 0.0001, respectively).

### CD 133 expression and TMZ treatment

**Fig 8:**
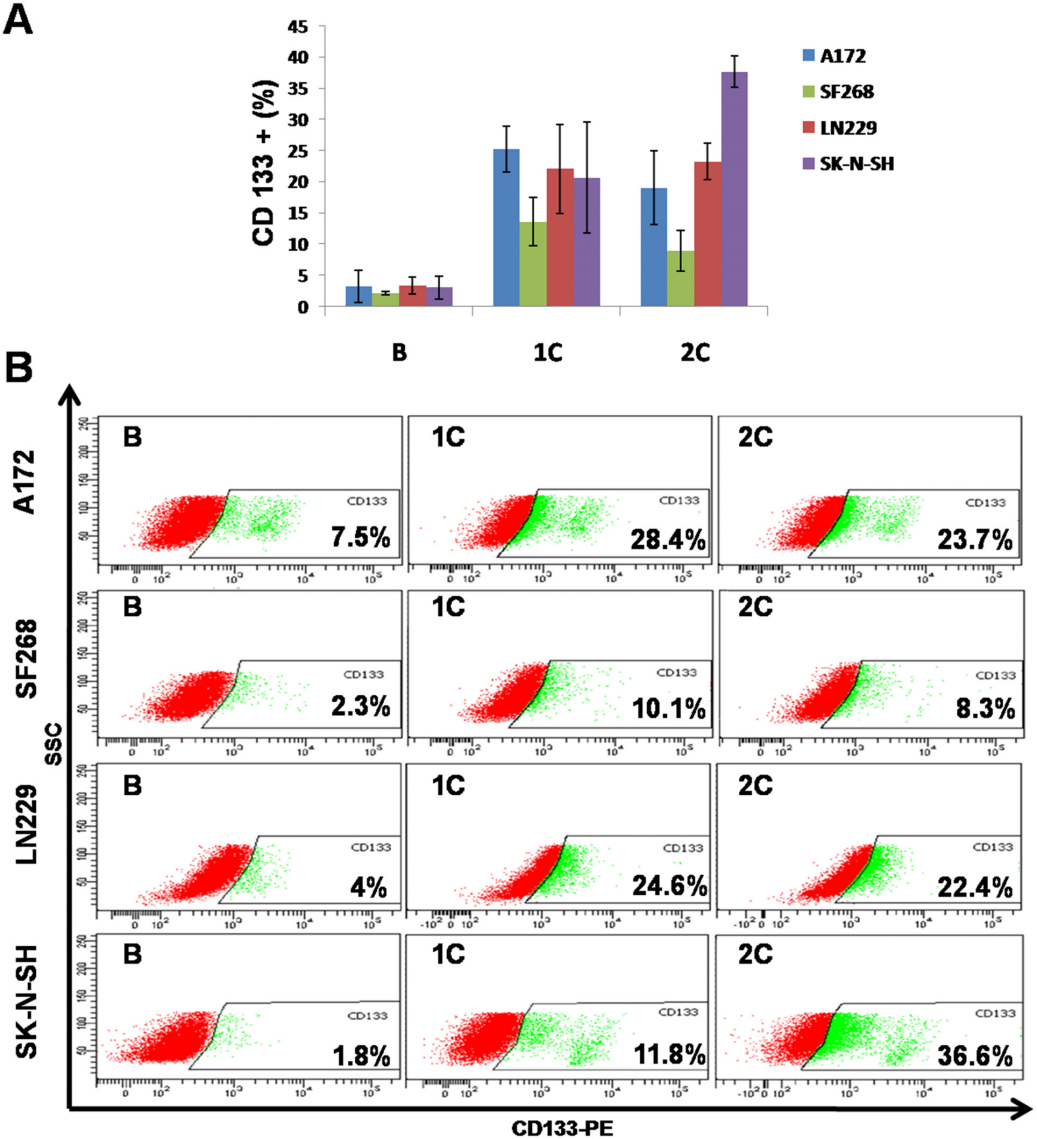
Modulation of CD 133 expression by TMZ treatment. A) Flow cytometry analysis of percentages of CD133 positives in tumor cell lines using TMZ treatment B: basal cells, 1C: first TMZ cycle; 2C: second TMZ cycle. Data were calculated from three independent experiments. B) Representative flow cytometry data for CD133 expression. SSC: side-scattered light.

In order to estimate the percentage of cancer stem cells in the cancer cell population after performing two TMZ cycles, the expression of the cancer stem cell marker CD133 was evaluated. Our results showed a low percentage of CD133 positive (CD133+) population in all cell lines under basal conditions (3.23 ± 2.57% in A172; 3.40 ± 1.40% in LN229; 2.13 ± 0.28% in SF268 and 3.06 ± 1.85% in SK-N-SH) (Fig 8). Interestingly, a significant increase in the CD133+ population was detected after the first TMZ cycle in all tumoral cell lines (25.23 ± 3.67% in A172; 22.13 ± 7.13% in LN229; 13.63 ± 3.82% in SF268 and 20.73 ± 8.90% in SK-N-SH). A further increase in CD133+ population was observed after the second TMZ cycle in SK-N-SH (37.67 ± 2.54%) while the other cells lines showed a slight decrease compared to the first cycle, but all of them maintained a CD133+ higher percentage than those observed at baseline (19.10 ± 5.93% in A172, 23.27 ± 2.90% in LN229 and 8.97 ± 3.25% in SF268). These results are statistically significant at A172, LN229 and SK-N-SH cell lines with a p value of 0.025, 0.016 and 0.001 respectively. The only cell line which shows a no significant p value is SF268 with a p value of 0.060. These results are similar to those obtained by determining CD133 expression by RealTime (S1 Fig).

## Discussion

Althought the use of TMZ represented a moderate improvement in the prognosis of GBM patients (a survival rate increase of around 20%), the development of drug resistance is one of the main causes of treatment failure [21]. To the molecular mechanism(s) underlying this drug resistance phenomenon remains unclear [22]. TMZ resistance has typically been related to the presence of the enzyme MGMT, with the modulation of MGMT expression appearing to be regulated by MGMT gene promoter methylation. Indeed, some *in vivo* studies have demonstrated TMZ treatment to be more effective when combined with a methylated MGMT gene promoter that induces protein silencing [11,23,24]. However, some studies have indicated that this correlation does not hold true in all cases. Thus, Hegi et al. [25] concluded that there is no clear relationship between patients with methylated MGMT promoter and a favorable response to TMZ treatment, and Yin et al. [26] determined the predictive but not prognostic value of MGMT promoter methylation status in elderly GBM patients after TMZ treatment. Other authors, such as Wick et al. [27], indicated the need to combine MGMT with other biomarkers. These contradictory findings indicate that the MGMT protein in GBM patients may not be the only DNA repair mechanism involved in the response to alkylating agents, with other repair mechanisms possibly being involved.

Our *in vitro* results reveal a significant association between MGMT expression in tumor cells and TMZ resistance. Indeed, SF268 and SK-N-SH cell lines, which are characterized by high levels of MGMT mRNA and protein expression, showed a pronounced resistance to TMZ (TMZ IC_50_ of 147.2 ± 2.1 μM and 234.6 ± 2.3 μM, respectively), whereas A172 and LN229 cell lines which exhibit no MGMT expression, both have low TMZ IC_50_ values (14.1 ± 1.1 μM and 14.5 ± 1.1 μM respectively). Similar results were observed by Gaspar et al. [28], who obtain TMZ IC_50_ values of between 10 and 20 μmol/L for A172 and LN229 cell lines, thereby establishing a direct correlation between MGMT methylation and TMZ sensitivity. Subsequent studies by Yoshino et al. [29] found that low MGMT expression levels in A172, U-87MG, and U-251MG cell lines correlated with low TMZ IC_50_ concentrations (< 100 μM), whereas high MGMT expression in T98G and U-138MG cell lines was associated with high IC_50_ values (> 350 μM). Interestingly, cell lines that do not express MGMT showed an exponential increase of TMZ resistance after two TMZ treatment cycles. Thus, the LN229 cell line, which has a low basal TMZ IC_50_ (14.5 ± 1.1 μM), has exhibited a TMZ IC_50_ of 109.4 ± 1.9 and 547.4 ± 2.6 μM after the first and second TMZ cycles, respectively. Conversely, cell lines with high levels of MGMT expression, such as SF268 and SK-N-SH, maintained their basal TMZ IC_50_ despite two TMZ cycles (234.6 ± 2.3 μM and 147.2 ± 2.1 μM, respectively). This phenomenon has also been described in the generation of resistant GBM cell lines [30,31].

The negative correlation between MGMT mRNA and protein levels in some cell lines such as SF268 and SK-N-SH supports the results of some authors [32,33] who described serious difficulty in correlating methylation and MGMT expression. It is known that MGMT protein is depleted by TMZ in a dose dependent form and that this enzyme is degraded by ubiquitination mechanisms. When the alkylating agent is added, the cell experiences an increasing demand for MGMT protein, generating a greater amount of mRNA which is quickly translated into protein. This phenomenon could explain the imbalance between MGMT mRNA and protein levels after TMZ exposure, although more experimentation is necessary to clarify the regulation of the MGMT expression mechanism.

After determining TMZ IC_50_ in tumor cells, we also analyzed the modulation of the cell cycle as this drug, like most DNA-damaging chemotherapeutic agents, induces cell cycle arrest [34]. Our results showed a G2/M blockage in A172 and LN299 cell lines after TMZ treatment (46% and 20%, respectively). In contrast, SF268 and SK-N-SH cell lines, both of which exhibit high TMZ resistance, showed no significant differences after TMZ exposure with respect to untreated cells. The relevance of these cell cycle modifications will be discussed later.

To determine the relationship between MGMT promoter gene methylation, MGMT expression, and TMZ treatment, we analyzed the methylation status of the promoter in all tumor cell lines before and after TMZ treatment. It is clear that cell lines with low TMZ IC_50_ concentrations experienced a high percentage of promoter methylation, whereas this percentage was much lower in cell lines with a high TMZ IC_50_. However, no significant changes in promoter methylation were detected in either the A172 or LN229 cell lines after TMZ treatment. Further to this, only small changes were noted in the SF268 cell line. SK-N-SH cells, with a methylation range of 75–100%, were the only cell line with a gradual loss of MGMT promoter methylation, decreasing to 25–50% after the first TMZ cycle and then 0–25% after the second. As stated above, it is difficult to correlate methylation status with MGMT expression. In fact, Fig 3 shows a significant increase in MGMT mRNA expression in A172 and LN229 cell lines without any variations in MGMT promoter methylation. This could be explained by the heterogeneity of the tumor cell lines which means different cell populations may have different methylation patterns and therefore different MGMT expression levels (including a very low MGMT amount). Hence the MGMT mRNA increase could be induced by some population cells without cell lines that express MGMT in the basal form. Therefore, no change in the methylation pattern could be observed, probably because it did not occur as such. Interestingly, analysis of MGMT expression showed a decrease in SF268 and SK-N-SH and a very small increase in A172 and LN229 cell lines. Thus, in light of these results, the correlation between methylation variation and MGMT expression levels remains unclear.

In this context, experiments were performed with 5-Aza and O6-BG. Treatment with 5-Aza, which can demethylate the MGMT promoter [35], increased MGMT expression in tumor cell lines. Interestingly, the TMZ IC_50_ for A172 and LN229 cell lines (with no basal MGMT expression) suffered a significant increase (from 14 ± 1.1 μM to 66 ± 1.6 μM p = < 0.0001), whereas no modulation of TMZ IC_50_ was observed for the SF268 and SK-N-SH lines, which have a high basal MGMT expression. Treatment with O6-BG, which is capable of blocking MGMT action [28], decreased MGMT protein levels in these latter cell lines, as shown by Western blot analysis, but did not induce significant changes in the TMZ IC_50_. Our results support the findings of Von Bueren et al. [36], who observed that TMZ resistance did not decrease in medulloblastoma cell lines after MGMT inhibition with O6-BG treatment, and contrast with that of Zhang et al. [37], who suggested that MGMT plays a critical role in TMZ resistance. As such, other resistance mechanisms could play a part in modulating the efficacy of TMZ in GBM cells.

The MMR complex is involved in repairing DNA damage caused by alkylating agents. Deficiencies in the expression of MSH2, MSH6, and PMS2, which form part of the MMR complex, have been linked to modulation of TMZ resistance independently of MGMT [30]. Indeed, Gaspar et al. [28] correlated a high MMR complex expression with a low TMZ IC_50_ in the GBM A172 cell line. In addition, Yip et al. [38] concluded that a decrease in MSH6 expression in a clone taken from the A172 cell line correlated with TMZ resistance in comparison to the sensitive A172 cell line which showed normal MSH6 expression. We analysed the presence of five of the most important genes forming the MMR complex (MLH1, MSH2, MSH3, MSH6, and PMS2) in our tumor cell lines before and after TMZ treatment. The results clearly showed that cell lines with a high MGMT expression level and a high TMZ IC_50_ had a very low level of MMR expression, whereas cell lines with no MGMT expression and a low TMZ IC_50_ were characterized by high MMR expression levels. In addition, TMZ exposure induced a general decrease in MMR expression in all tumor cells, with this decrease being most pronounced in the A172 cell line (more than 80%). Although TMZ treatment increased expression of MSH3, MSH6, and PMS2 in SF268 and SK-N-SH cell lines, the significant decrease in MLH1 and MSH2 expression detected could explain the observed deficiency in MMR function. It is known that MLH1 protein binds the MMR complex with to DNA after mismatch recognition [39] and that MSH2 plays an important role in stabilizing MLH1 [40]. Interestingly, recent studies demonstrated a strong association between some MMR complex subunits (such as MLH1 expression), tumor recurrence, and TMZ resistance in GBM cell lines [38]. Thus, the inability of TMZ cycles to modulate the IC_50_ in SF268 and SK-N-SH cell lines could be related to a compensatory mechanism in which the decrease in MGMT expression (which increases the cell's sensitivity to TMZ) is accompanied by a decrease in the levels of MMR complex, thereby further increasing the cell’s resistance to TMZ. This could refute the classical concepts in which TMZ resistance is only mediated by MGMT expression since a blockade of MGMT does not affect the sensitivity to TMZ. Maxwell et al. [41] showed that patients with a high TMZ resistance have an altered MMR function, as determined by the reduced expression of the MSH6 subunit. Hirose et al. [34] demonstrated that, in the GBM U87MG cell line (p53 wild-type phenotype), TMZ at 100 μM induced p53-dependent G2/M cell cycle arrest but did not induce MGMT expression. This cell line showed similarities to the LN229 line. In contrast, the A172 cell line (p53 mutated phenotype) has no pro-apoptotic activity [42]. This fact could explain why A172 cells that presented low MMR expression levels after two TMZ cycles (A172-2C) do not experience as much blockage in the G2/M phase, as alternative apoptosis could occur in the p73 pathway [42]. On the other hand, the SF268 cell line (resistant to TMZ) showed G2/M phase accumulation with no differences between basal and treated cells. This could be justified by its mutated p53 phenotype and low level of MMR expression which prevents the p73 apoptosis pathway [8]. SK-N-SH presented a p53 wild-type phenotype and a blockage in the G2/M phase after TMZ treatment, but with no cell cycle modulation after drug exposure [8]. As with SF268, the G2/M phase blockage may be related to the lower levels of MMR, which is responsible for recognizing GT mismatches and triggering cell cycle arrest, cellular senescence, and apoptosis in TMZ-treated cells [8].

Although P-gp has recently been associated with nervous system tumors [22,43], our analyses showed no P-gp expression in either of the tumor cell lines studied, in agreement with the limited data available with respect to GBM lines [44]. However, TMZ exposure increased P-gp expression levels in all cell lines. To test the relevance of this molecule to resistance mechanisms in GBM cells, we applied a TMZ treatment before and after verapamil exposure [45]. No changes in TMZ IC_50_ were observed for A172 and LN229 cell lines (p = 0.3875). In contrast, treatment with verapamil significantly increased the TMZ IC_50_ values for SF268 and SK-N-SH (p = 0.0379 and p = < 0.0001, respectively). This seemingly contradictory result can be explained by verapamil's ability to inhibit membrane calcium transporters [46]. In cells with low levels of the MMR system (SF268 and SK-N-SH) apoptosis was mediated by intramitochondrial-calcium-dependent pathways (PARP, calpaina and AIF (parthanatos)) [47], thus meaning that verapamil could block cell death. Indeed, A172 and LN229 cell lines (with high MMR levels) did not reveal any TMZ IC_50_ modulation. Thus, P-gp does not appear to be related to TMZ resistance in GBM patients.

Finally, the theory in which cytotoxic treatment selects resistant CSCs could explain post-therapy cancer drug resistance. Although CSCs may be detected in different tumors, including GBM, by the presence of CD133 [19], the value of the latter as a prognostic parameter is unclear. Several *in vivo* studies have demonstrated that the presence of CSCs (CD133 positive) in GBM correlated with chemoradioresistance and a poor prognosis [48], whereas Melguizo et al. [11] recently demonstrated that CD133 has no implication in the prognosis of GBM patients, supporting similar findings reported by Kim et al. [49]. Some authors even correlated high expression of CD133 with a better GBM prognosis [50]. In this context, we studied CD133+ population in our cell lines before and after TMZ treatment. CD133 expression has been associated with drug resistance in SK-N-SH [51] and A172 cell lines [52]. Our results showed a significant increase in the percentage of CD133+ cells after the first TMZ cycle in A172 and LN229 cell lines. Interestingly, after treatment, both cell lines also showed a major increase in the TMZ IC50. This CD133 population could make a moderate contribution to increasing TMZ resistance during the treatment. On the other hand, the low percentage of CD133+ cell population in SF268 and SK-N-SH basal cell lines could be indicative of an unclear correlation between this population and initial TMZ resistance. However, TMZ caused an clear enrichment of this population, suggesting that TMZ cycles treatment promoted the generation of cancer stem cells.

## Conclusions

The molecular mechanisms mediating temozolomide resistance, one of the leading causes of treatment failure in GBM patients, are unclear. As such, we used nervous system tumor cell lines exposed to TMZ treatment to analyze the relevance of MGMT, the MMR system, and the ABC transporter in the TMZ resistance phenomenon. Our results demonstrate that MGMT does not play a key role in TMZ resistance in either A172 or LN229 cell lines (both with low MGMT expression levels), and nor does it have an essential part in either SF268 or SK-N-SH lines, both of which have high levels of MGMT expression. It is worth noting, however, that the degree of MGMT expression correlates with expression of the MMR complex, which is also modulated by TMZ. This may have a bearing on TMZ resistance and could explain the failure of TMZ treatment in tumor cells with no MGMT expression. In contrast, the ABC-transport protein P-gp does not appear to participate in TMZ drug resistance. Furthermore, we have demonstrated that TMZ treatment produces a significant change in CD133+ cell percentages in GMB cell lines. Further studies into CD133+ population characteristics in GMB cell lines are necessary to determine the population's relevance to drug resistance and its differences with respect to the negative CD133 cell population. Thus, therapeutic strategies that restore the expression of the MMR system could lead to possible routes to improve the efficacy of TMZ therapy in GBM patients.

## Supporting Information

S1 Fig Modulation of CD 133 expression by TMZ treatment. Real-time PCR analysis. A) Real-time PCR analysis of CD133 expression in tumor cell lines. The A549 cell line was used as a positive control. B) Modulation of CD133 expression in tumor cell lines by TMZ treatment. B: basal cells, 1C: first TMZ cycle; 2C: second TMZ cycle. All data represent the mean value ± SD of triplicate cultures.
(TIF)

## Abbreviations

TMZ: Temozolomide
GBM: Glioblastoma Multiforme
MGMT: O6-methylguanine-DNA methyltransferase
MMR: Mismatch repair
P-gp: P-glycoprotein
5-Aza: 5-Aza-2′-deoxycytidine
O6-BG: O(6)-benzylguanine
CSC: Cancer stem cells
MSP: Methylation-specific PCR
M: Methylated
UM: Unmethylated
