# Supplementary figures and images for: Temozolomide Resistance in Glioblastoma Cell Lines: Implication of MGMT, MMR, P-Glycoprotein and CD133 Expression
Source: PLoS One. 2015 Oct 8;10(10):e0140131. doi: 10.1371/journal.pone.0140131 (PMC4598115; doi:10.1371/journal.pone.0140131)

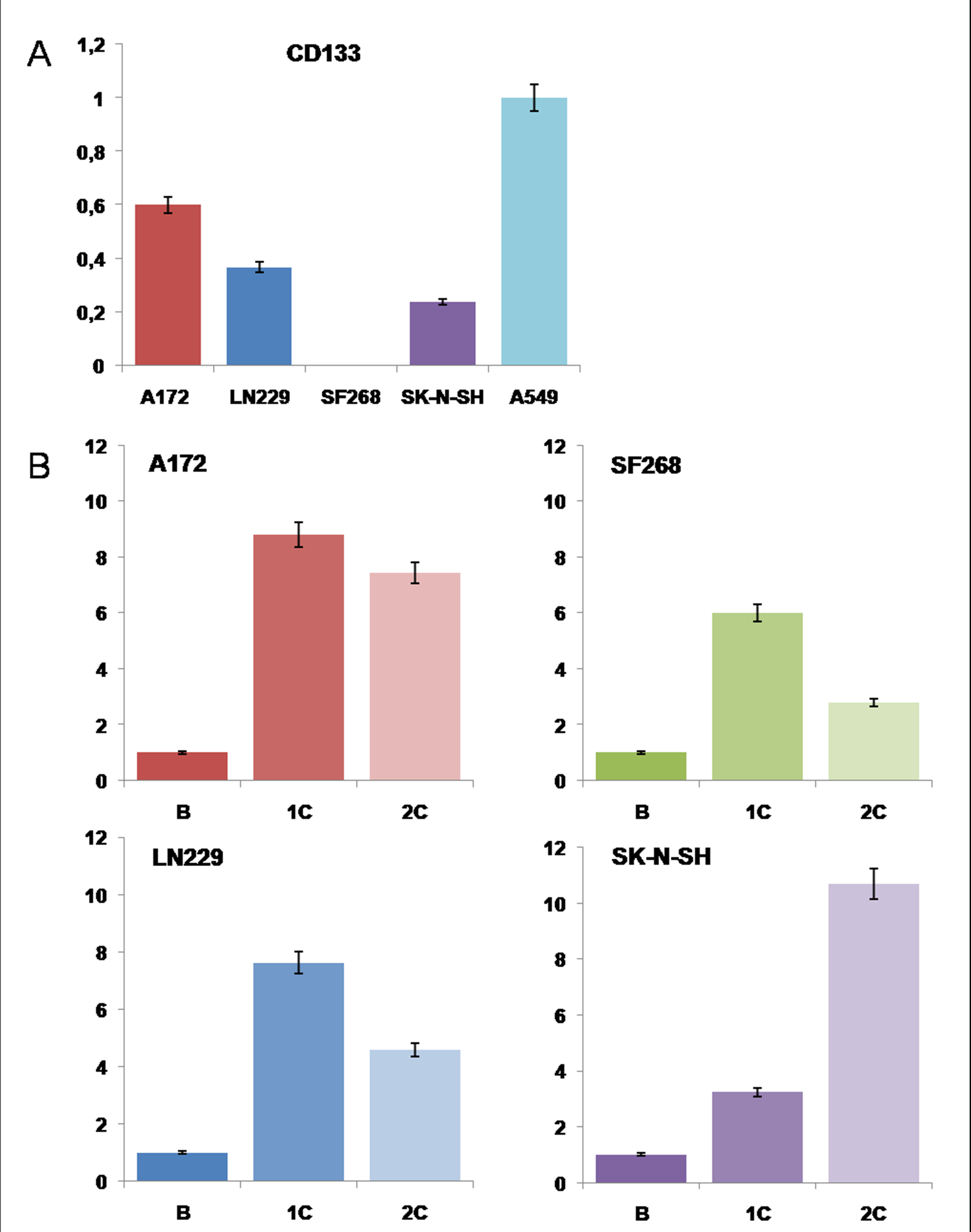

Supplement: S1 Fig — A) Real-time PCR analysis of CD133 expression in tumor cell lines. The A549 cell line was used as a positive control. B) Modulation of CD133 expression in tumor cell lines by TMZ treatment. B: basal cells, 1C: first TMZ cycle; 2C: second TMZ cycle. All data represent the mean value ± SD of triplicate cultures. (TIF) [file pone.0140131.s001.tif]
